# Estimated Pulse Wave Velocity and All-Cause and Cardiovascular Mortality in the General Population

**DOI:** 10.3390/jcm13123377

**Published:** 2024-06-07

**Authors:** Vladimir Prelević, Luka Blagus, Vito Bošnjak, Danilo Radunović, Mihaela Marinović Glavić, Vedran Premužić, Jelena Kos, Ivan Pećin, Tajana Željković Vrkić, Marija Domislović, Ana Jelaković, Viktor Domislović, Krunoslav Capak, Marija Bubaš, Valentina Kriksić, Bojan Jelaković

**Affiliations:** 1 Department of Nephrology, Hypertension, Dialysis and Transplantation, University Hospital Center Zagreb, 10000 Zagreb, Croatia; danilo.radunovic@kccg.me (D.R.); vpremuzic@gmail.com (V.P.); jkos@kbc-zagreb.hr (J.K.); marija.domislovic@kbc-zagreb.hr (M.D.); anajelakovic9@gmail.com (A.J.); jelakovicbojan@gmail.com (B.J.); 2 Clinic for Nephrology, Clinical Center of Montenegro, 81000 Podgorica, Montenegro; 3 Family Medicine, Health Center Zagreb-Centar, 10000 Zagreb, Croatia; luka.blagus2606@gmail.com; 4 Department for Plastic, Reconstructive and Aesthetic Surgery, University Hospital “Sveti Duh”, 10000 Zagreb, Croatia; vbosnjak@kbsd.hr; 5 School of Medicine, University of Rijeka, 51000 Rijeka, Croatia; mihaela.marinovic@mediri.uniri.hr; 6 Department of Metabolic Diseases, University Hospital Center Zagreb, 10000 Zagreb, Croatia; ipecin@kbc-zagreb.hr; 7 School of Medicine, University of Zagreb, 10000 Zagreb, Croatia; 8 Department for Cardiovascular Diseases, Institution for Cardiovascular Prevention and Rehabilitation, 10000 Zagreb, Croatia; tajana.zeljkovic.vrkic@kbc-zagreb.hr; 9 Department of Gastroenterology and Hepatology, University Hospital Center Zagreb, 10000 Zagreb, Croatia; viktor.domislovic@gmail.com; 10 Croatian Public Health Institute, 10000 Zagreb, Croatia; krunoslav.capak@hzjz.hr (K.C.); marija.bubas@hzjz.hr (M.B.); 11 University North, 48000 Koprivnica, Croatia; 12 Institution for Home Health Care “Dominus”, 10000 Zagreb, Croatia; valentina@domnius.hr

**Keywords:** arterial stiffness, estimated pulse wave velocity, cardiovascular mortality, all-cause mortality

## Abstract

**Background:** Carotid-femoral pulse wave velocity (cfPWV), acknowledged as a reliable proxy of arterial stiffness, is an independent predictor of cardiovascular (CV) events. Carotid-femoral PWV is considered the gold standard for the estimation of arterial stiffness. cfPWV is a demanding, time consuming and expensive method, and an estimated PWV (ePWV) has been suggested as an alternative method when cfPWV is not available. Our aim was to analyze the predictive role of ePWV for CV and all-cause mortality in the general population. **Methods:** In a stratified random sample of 1086 subjects from the general Croatian adult population (EH-UH study) (men 42.4%, average age 53 ± 16), subjects were followed for 17 years. ePWV was calculated using the following formula: ePWV = 9.587 − 0.402 × age + 4.560 × 10^−3^ × age2 − 2.621 × 10^−5^ × age2 × MBP + 3.176 × 10^−3^ × age × MBP − 1.832 × 10^−2^ × MBP. MBP= (DBP) + 0.4(SBP − DBP). **Results:** At the end of the follow-up period, there were 228 deaths (CV, stroke, cancer, dementia and degenerative diseases, COLD, and others 43.4%, 10.5%, 28.5%, 5.2%, 3.1%, 9.3%, respectively). In the third ePWV tercile, we observed more deaths due to CV disease than to cancer (20.5% vs. 51.04%). In a Cox regression analysis, for each increase in ePWV of 1 m/s, there was a 14% increase risk for CV death. In the subgroup of subjects with higher CV risk, we found ePWV to be a significant predictor of CV deaths (ePWV (m/s) CI 1.108; *p* < 0.029; HR 3.03, 95% CI 1.118–8.211). **Conclusions:** In subjects with high CV risk, ePWV was a significant and independent predictor of CV mortality.

## 1. Introduction

Arterial stiffness measured directly and non-invasively by carotid-femoral pulse wave velocity (cf PWV) is an important biomarker of cardiovascular (CV) health, and it predicts CV events beyond and independently of traditional risk factors [1,2,3,4,5,6,7]. Carotid-femoral PWV is considered the gold-standard method for the estimation of arterial stiffness. Based on these facts, some authors have proposed that the assessment of arterial stiffness, in addition to the assessment of albuminuria and left ventricular hypertrophy, should be included in clinical evaluations of target-organ damage [1,2,3,4,7,8]. It has been suggested that increased cfPWV is a reliable biomarker of CV and mortality risk [8,9,10,11,12]. It has also been proposed that measurement using cfPWV could guide the tailoring of drug doses [1,3,8]. However, cfPWV is a demanding, time-consuming and expensive method, which is frequently not available; therefore, its use in clinical practice is rather rare. Recently, Greeve at al. reported that an estimated pulse wave velocity (ePWV) can be calculated from age and mean BP using the quadratic equation generated from the Reference Values for Arterial Stiffness Collaboration [13]. They found that ePWV predicted a combined CV end point independently of Systemic Coronary Risk Evaluation (SCORE), Framingham risk score (FRS), and cfPWV. This result was confirmed by Vlachopoulos et al. in very high-risk hypertensive patients (the SPRINT study), showing that ePWV predicted the primary composite CV outcome and all-cause death independent of the FRS [14]. Later on, observations that ePWV predicted all-cause and CV mortality independently of traditional CV risk factors were reported in the general population, apparently healthy individuals and high-risk patients [8,14,15,16,17,18,19,20,21,22,23,24,25,26,27,28,29,30,31]. However, in most of these studies, several groups of subjects were excluded, so the results could not be extrapolated to the general population. Evidence on the predictive value of ePWV on all-cause and CV mortality in a community-based general population is extremely scarce, and it is an inexpensive and easily attained measure of vascular age. Those facts increase the temptation to use ePWV as a replacement for cfPWV. However, the debate is focused on the fact that real measurements using cfPWV could not be sufficiently replaced with estimated, and not real time-proven, PWV [32]. 

Arterial stiffness can be also assessed with carotid ultrasonography, and it offers high clinical value in many clinical scenarios. The ultrasonographically assessed resistance index is independent from heart rate and is an objective cardiovascular risk factor for adverse events [33].

The main purpose of this study was to investigate the predictive role of ePWV in CV and overall mortality in the general population with arterial hypertension in the random representative nationwide sample of Republic of Croatia during a follow- up period of 17 years. 

## 2. Materials and Methods

Population: In this observational, prospective, nationwide study (Epidemiology of Hypertension in Croatia, EH-UH study), subjects over the age of 18 were randomly selected from the general population using a series of randomized numbers that represented the ordinal number of the insured in the registers and documentation of family physicians. The participation rate was 70.2%. A total of 1086 subjects (460 men, 626 women) were included. The inclusion criteria were: (a) age over 18 years; (b) signed informed consent form. The exclusion criteria were: (a) pregnancy and lactation; (b) terminal illness and life expectancy less than 6 months; (c) dementia or cognitive dysfunction; (d) amputation of one or more extremities; (e) other restrictions that prevent the implementation of the protocol (paresis, limb amputation, immobilization of one of the hands due to trauma); (f) unsigned consent form. Patients with diabetes or those who suffered myocardial infarction or stroke before 3 months were included. Body mass index (BMI) was calculated, and according to the BMI value, the subjects were divided into three categories: ≥30 kg/m^2^ obesity; 25–30 kg/m^2^ overweight; and <25 kg/m^2^ normal body weight. Hypertension was defined as BP ≥ 140/90 mmHg and/or taking antihypertensive therapy. Diabetes mellitus was defined as antidiabetic therapy and/or fasting glucose > 7 mmol/L. The estimated glomerular filtration rate (eGFR) was calculated using the CKD-EPI equation.

The study was performed in accordance with the standard of the Declaration of Helsinki. 

The study protocol was reviewed and approved by the Ethic Committee of School of Medicine University of Zagreb, approval number 587. 

Procedure: During the visit, the respondents signed a written consent form, after which an interview was conducted (structured questionnaire), and a clinical exam and measurements were performed. A standardized questionnaire included questions on demographic, socioeconomic and clinical parameters. BP was measured with a standard mercury sphygmomanometer that had been calibrated prior to the study, and with a cuff of appropriate size. BP was measured in sitting position after a five-minute rest, first on both arms in a sitting position, and then on the arm with higher systolic BP. From the sum of the second and third measurement, the average value of BP was calculated, which was later used in statistical processing. After each BP measurement, the heart rate was measured by palpation of the radial artery at intervals of 30 s. We measured anthropometric parameters; the subject’s body height (cm) and weight (kg) were measured without shoes in light clothing. Decades were defined as ≤30 years, 31–40 years, 41–50 years, 51–60 years, 61–70 years, 71–80 years, and 81–90 years. 

Estimated pulse wave velocity (ePWV) was calculated using a validated equation descrined by Greve et al. derived by the Reference Values for Arterial Stiffness’ Collaboration [13]: (a) for individuals with CV risk factors: ePWV = 9.587 − 0.402 × age + 4.560 × 10^−3^ × age^2^ − 2.621 × 10^−5^ × age^2^ × mean AT + 3.176 × 10^−3^ × age × mean AT − 1.832 × 10^−2^ × medium AT; for subjects without CV risk factors: ePWV as: ePWV = 4.62 − 0.13*age + 0.0018*age^2^ + 0.0006*age*MBP + 0.0284*MBP. Individuals without CV risk factors were defined as non-smokers without any components of a metabolic syndrome and without a history of myocardial infarction or stroke.

Mean BP = DBP + 0.4 × (SBP − DBP).

Form factor 0.4 was used, since it has been demonstrated that mean BP calculation using 0.4 is superior for the discrimination of subjects with left ventricular and carotid wall hypertrophy, as well as subjects with increased aortic stiffness [34].

ePWV were categorized according to terciles. 

For the better assessment of mortality in elderly people, total arterial compliance was calculated.

Total arterial compliance was calculated with formula:

Ct = k × PWV

(where factor k = 37 for BMI 26.2 kg/m^2^) [35]. 

Mortality data: Mortality data were obtained from the records of the Croatian Institute of Public Health. In the follow-up period of 17 years, in our group, 233 deaths were recorded. We excluded six individuals who died within the first 12 months of the follow-up period to address potential concerns with reverse causality. Furthermore, we excluded seventeen subjects whose death was not marked according to the International Classification of Diseases and Related Health Problems ICD-10 and eight subjects without exact data on the date of death. Finally, in the group of 1060 subjects with all requested information, there were a total of 202 deaths (19%). CV mortality consisted of fatal stroke, fatal myocardial infarction, or coronary death. All-cause mortality was also assessed. 

Statistical data processing: The data of the categorical variables are presented as number (n) and percentages (%). The data of the continuous variables are presented as the mean and SD (standard deviation) and as the median and corresponding 25th and 75th percentile for skewed variables. The comparison of continuous variables between individual groups was performed using Student’s *t* test and the ANOVA test, and the comparison of categorical variables between individual groups was performed using the Chi-square test. Univariate and multivariate linear regression methods were used to determine ePWV-related factors. Kaplan–Meier curves and log-rank test were used to compare survival times and cumulative incidence of CV and all-cause mortality among groups of subjects classified into the terciles according to ePWV values. The associations of ePWV with CV and all-cause mortality were determined by Cox proportional hazards regression models with or without adjustment for the selected confounders. 

Model 1 was an unadjusted model, while model 2 was adjusted for age, gender, BMI, mean BP and HR, and model 3 was adjusted for diabetes and HR.

The prognostic value of ePWV was analyzed by a clinically relevant cut-off. To this purpose, a time-dependent survival receiver operating characteristic curve (ROC) was implemented using Kaplan–Meier estimates and identified a cut-off point that optimized the combination of sensitivity (true-positive) and 1-specificity (false-positive). The Youden index method was used to calculate optimal cut-off levels. Statistical calculations were performed by SPSS statistical software (IBM^®^ SPSS^®^, version 26). We deemed statistical significance at α = 0.05.

## 3. Results

The average age and BMI of the whole group (42.4% men) were 53 ± 16 years, 27.2 ± 4.8 kg/m^2^, respectively (Table S1). Hypertension, diabetes, and chronic kidney disease were diagnosed in 46.9%, 9.4% and 7.8%, respectively (Tables S1 and S2). The average ePWV in the whole group was 9.59 ± 2.52 m/s. Mortality events were documented during 17 years of follow up. When analyzing the proportion of individual causes of death in the group of 202 subjects who died, 43.06%, 28.7%, 10.9% and 17.8% referred to the CV disease, cancer, stroke, and to other causes, respectively. In univariate logistic regression, predictors of all-cause deaths were higher ePWV, older age, lower body height, higher BMI, higher systolic, diastolic and mean BP, faster heart rate, higher fasting blood glucose, total cholesterol, uric acid, and lower eGFR (Table S4). Predictors of CV deaths were higher ePWV, older age, lower body height, higher BMI, higher systolic, diastolic, and mean BP, higher fasting blood glucose, uric acid, and lower eGFR (Table S5). 

Characteristics of subjects divided into the ePWV terciles are shown in Table 1. Subjects in the third ePWV tercile were the oldest, with the highest BMI, BP and heart rate. They had the significantly highest values of fasting blood glucose, total cholesterol, LDL-cholesterol, and uric acid and the lowest values of eGFR. Dividing the subjects into ePWV terciles, the highest number of deaths was in the third tercile (69.8%), and the lowest was in the first tercile (5.4%). Interestingly, we observed a difference in frequency of cancer and CV deaths between the second tercile and third tercile (46% and 30% vs. 20.5% and 51.0%, respectively, *p* < 0.001). A detailed list of causes of death by terciles is given in the Supplementary Materials (Table S5). 

**Figure 1:**
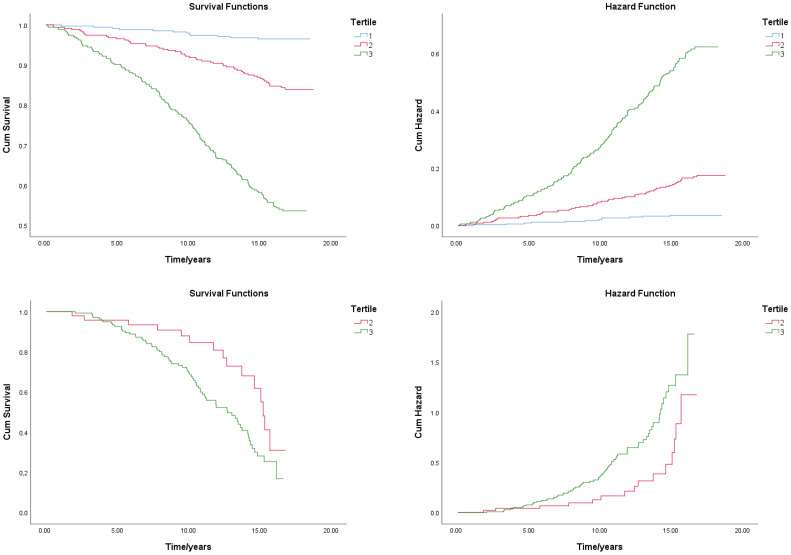
Kaplan–Meier curves comparing the cumulative incidence of all-cause and cardiovascular mortality in subjects classified in ePWV terciles. First row—all-cause death; second row—CV death.

When CV mortality was observed, the probability of survival of those in the third tercile compared to the second tercile was statistically significantly lower (*p* = 0.013 Log Rank test (Mantel-Cox)). Figure 1 shows the Kaplan–Meier survival curves of subjects classified by terciles. In the Cox regression analysis, ePWV and Ct were significantly associated with CV and all-cause deaths (Table 2 and Table 3). In model 2, adjusted for age, gender, BMI, and mean BP, using ePWV, HR was significantly associated with all-cause mortality but not with CV mortality. In the analysis of survival of subjects classified into the terciles according to the ePWV values when all-cause death mortality was observed, the probability of survival of those in the third tercile compared to the second and the first tercile was statistically significantly lower (*p* < 0.001 log rank test (Mantel–Cox)) (Table 4). 

**Figure 2:**
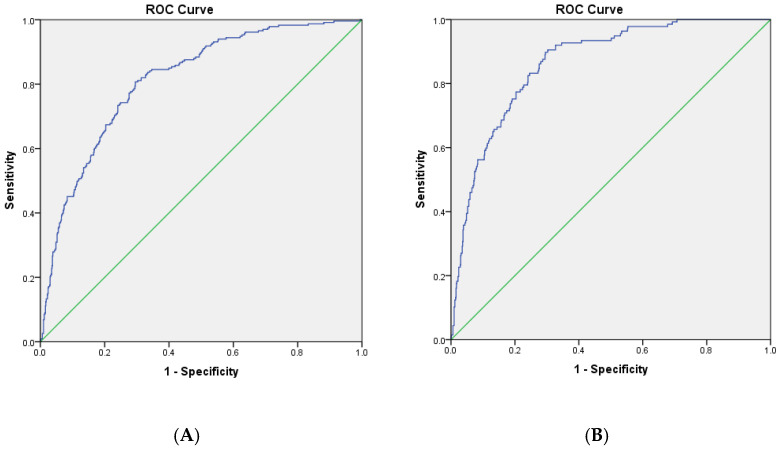
Area under the ROC (receiver operator characteristic) curve of ePWV and all-cause (**A**) and cardiovascular (**B**) mortality.

An increase of 1 m/s resulted in three times higher risk for CV mortality. In the model 3 using ePWV, adjusted for diabetes, ePWV was a significant predictor of CV deaths. When analyzing a subgroup of subjects with higher CV risk, those with metabolic syndrome or smokers, we found ePWV to be a significant predictor for CV death. Survival ROC analysis demonstrated that the optimal cut point for ePWV to discriminate CV, all-cause and non-CV mortality status were 10.38 m/s, 10.65 m/s and 10.96 m/s, respectively (Figure 2 and Table 4). 

## 4. Discussion

In this study, we examined the association between ePWV, a suggested proxy of vascular aging, and all-cause and CV mortality in a nationally representative cohort of general adult population in Croatia. The main findings of our study are that in an unadjusted model, ePWV significantly predicted all-cause and CV mortality, and for each increase of 1 m/s, there was a 14% increased risk of CV death. In an adjusted model, ePWV remained to be a significant predictor for all-cause mortality (HR 1.90). Our observation is in line with the results of Ji et al. in Chinese men where each ePWV increase by 1 m/s increased risk for CV death and all-cause death by 22% and 10%, respectively [15]. In a group of middle-aged Caucasian people from Finland, Jae et al. found that, independently of traditional CV risk factors, the highest levels of ePWV were significantly associated with an increased risk for all-cause mortality (HR 1.39) and CV mortality (HR 1.79) as compared with the lowest level of ePWV [16]. Heffernan et al., after adjusting for age and BP, noticed an even higher increase in CV and all-cause mortality in the US population (47% and 52%, respectively) [21]. ePWV was associated with all-cause mortality, irrespective of hypertension status, but it was a predictor of CV mortality only in treated hypertensive patients, supporting the findings from Vlachopoulos et al. that ePWV may be a stronger predictor of CV outcomes in adults with higher CV risk [14]. This is in line with our result, while in a subgroup of high-risk patients, we found ePWV to be a significant predictor of CV mortality even after adjustment (HR 3.03), and this remained significantly associated with CV mortality in our model with diabetes. Our result is in concordance with results published by other authors who found that the risk of most cause-specific mortality increased from 53% to 102% for every 1 m/s increase in ePWV [30]. In a high-risk group of patients, Hsu et al. also found a high risk for CV mortality (HR 2.321) [18]. Vlacholopulous et al., in the SPRINT trial population of hypertensive patients with very high risk but without subjects with diabetes and those with positive history for stroke, found that ePWV was associated with all-cause death, CV death and non-CV death (HR, 1.65, HR, 1.39, HR, 1.76, respectively) independent of the FRS and other relevant confounders [14]. Analyzing the stroke population from the NHANES study, Huang et al. concluded that with an increase in ePWV of 1 m/s, the risk of all-cause and cardio-cerebrovascular mortality are increased by 44–57% and 47–72%, respectively [31]. On the contrary, in the apparently healthy European population included in the MORGAM Prospective Cohort Project, ePWV did not predict CV morbidity or mortality independently of traditional CV risk factors. They found that ePWV was predictive of all-cause mortality even after adjusting for all the traditional CV risk factors, which is, again, in concordance with our result. Rui et al. reported that the association between ePWV and all-cause mortality is more pronounced in women, never-smokers and non-diabetics [36]. It seems that the predictive value of ePWV for CV and all-cause mortality is not the same in low- and high-risk populations. According to the results of the majority of authors, in low-risk populations, ePWV independently predicted all-cause mortality. However, it did not prove to be an independent predictor for CV mortality. On the contrary, in high-risk populations, ePWV was found to be an independent predictor of CV mortality, while inconsistencies were found in reports on its independent predictive value for all-cause mortality. Vishram-Nielsen et al. suggested that ePWV is more than a marker of CV risk than is supported by findings, and that ePWV was associated with higher risk for residual mortality [8,14]. Heffernan et al. observed that, for every 1 m/s increase in ePWV, there was a 17% increased risk of residual-specific mortality [20]. In multivariate Cox regression models in our study, heart rate was not a statistically significant confounder associated with mortality rate in general and high-CV-risk populations, which is not in line with other studies [37,38,39]. They raised the question of whether ePWV may be considered a simple tool in risk assessment for CV and non-CV deaths in the general population. 

For the better assessment of mortality in elderly patients, we calculated total arterial compliance. A statically significant importance of Ct was found in the models, whereas confounders were age, sex, MBP, BMI and diabetes for CV mortality in general and high-CV-risk populations. This finding is in line with the results of the PROTEGER study [40]. 

Comparing ePWV and arterial compliance in the assessment of CV and all-cause mortality, it has been found that arterial compliance is a better predictor of CV mortality in the general population and in subgroups with high cardiovascular risk, while ePWV is better predictor of all-cause mortality in the general population. This may be a novel finding of this study. 

Our study has several limitations. First, we used only basal levels of BP and cfPWV was not measured. This is an observational study, so the association between ePWV and mortality cannot be interpreted as a causal relationship. Second, during the follow-up period, some patients may have started using antihypertensive/antilipemic/antidiabetic drugs, and some may have quit or started smoking. This was an epidemiological study with a risk of unmeasured residual confounding. Our study was aimed to evaluate mortality events, so nonfatal events were not studied. 

The strengths of our study include the enrollment of a large-scale heterogeneous general population, a national representative sample of adults with broad age spectrum and an almost equal proportion of men and women with standardized baseline assessment. The period of follow-up was long, with a large number of deaths. Individuals who died within the first 12 months of the follow-up period were excluded from the analyses to avoid reverse causality. This is one of the first and only studies of association between ePWV with CV risk and mortality in the general population, which compare ePWV and arterial compliance in the assessment of CV risk and mortality, especially for the population of this region of Europe, which may have an important scientific contribution and may be the novelty of this study. 

## 5. Conclusions

In an adult general population, we found an independent association between ePWV and the risk of all-cause mortality, which is in line with reports from other studies [14,20,21]. We observed an independent association of ePWV with CV mortality only in high-risk subgroups, which is similar to the results of other authors who analyzed high-risk patients [14,15,16,17,18,19,20,21]. Taken together, these observations suggest that ePWV is not only an indicator of age and BP effects on mortality but a useful measure of vascular aging.

The main finding of this research is that ePWV is strongly associated with CV mortality in high-risk patients in the general population. 

## Figures and Tables

**Table 1:** Demographic and clinical characteristics of subjects classified according to the ePWV terciles.

	1st Tercile	2nd Tercile	3rd Tercile	*p*
Age, (years)	36 ± 8 36 (29–42)	53 ± 7 48 (58–70)	70 ± 7 70 (66–74)	<0.001
Men (%)	42.7	44.4	39.8	0.58
Height, (cm)	1.72 ± 0.10 1.71 (1.64–1.79)	1.69 ± 0.9 1.68 (1.62–1.72)	1.66 ± 0.09 1.65 (1.59–1.72)	<0.001
Weight, (kg)	73.2 ± 14.7 72.0 (62.0–82.0)	80.1 ± 14.1 80.0 (70.0–89.0)	79.3 ± 14.4 78.0 (69.0–86.5)	<0.001
Body mass index, (kg/m^2^)	24.7 ± 3.9 24 (22.0–27.0)	27.9 ± 4.3 27.0 (25.0–31.0)	29.0 ± 5.2 28.0 (26.0–32.0)	<0.001
Systolic BP (mmHg)	118 ± 12 118 (111–125)	135 ± 14 132 (125–142)	152 ± 19 152 (138–165)	<0.001
Diastolic BP (mmHg)	77 ± 7 77 (72–81)	85 ± 8 83 (80–90)	89 ± 11 89 (81–96)	<0.001
Mean BP (mmHg)	93 ± 8 94 (88–98)	105 ± 10 103 (99–111)	114 ± 13 114 (105–123)	<0.001
Heart rate (bpm)	72 ± 8 71 (67–76)	73 ± 9 73 (67–79)	74 ± 9 74 (67–80)	0.032
Fasting blood glucose (mmol/L)	5.1 ± 0.7 5.1 (4.7–5.5)	5.8 ± 1.4 5.5 (4.9–6.1)	6.3 ± 2.2 5.7 (5.1–6.5)	<0.001
Serum creatinine, (μmol/L)	85 ± 14 87 (74–95)	86 ± 37 81 (75–91)	89 ± 27 86 (75–98)	0.455
eGFR (ml/min/1.73 m^2^)	89.0 ± 13.9 89.7 (78.4–95.3)	80.1 ± 18.1 80.8 (69.9–91.1)	66.5 ± 15.5 65.7 (54.6–77.5)	<0.001
Total cholesterol, (mmol/L)	5.6 ± 1.3 5.4 (4.8–6.5)	6.0 ± 1.2 6.0 (5.3 –6.6)	6.3 ± 1.3 6.1 (5.4–7.1)	<0.001
LDL cholesterol, (mmol/L)	3.7 ± 1.1 3.7 (2.8–4.6)	3.9 ± 1.0 3.9 (3.3–4.4)	4.1 ± 1.3 4.0 (3.1–4.9)	0.274
HDL cholesterol, (mmol/L)	1.4 ± 0.5 1.4 (1.1–1.6)	1.3 ± 0.4 1.3 (1.0–1.5)	1.4 ± 0.5 1.4 (1.1–1.6)	0.203
Triglycerides, (mmol/L)	1.5 ± 1.0 1.3 (0.9–1.8)	1.9 ± 1.2 1.6 (1.2–2.3)	1.9 ± 1.2 1.7 (1.1–2.3)	0.011
Uric acid, (mmol/L)	259.5 ± 87.0 239.0 (196.0–302.5)	286.3 ± 102.3 280.0 (213.0–326.0)	305.6 ± 84.3 297.0 (253.0–358.0)	0.004
ePWV (m/s)	6.9 ± 0.5 6.9 (6.5–7.4)	9.2 ± 0.9 9.1 (8.3–10.1)	12.6 ± 1.2 12.5 (11.6–13.4)	<0.001
Cancer deaths	1.7 (6)	6.3 (22)	8.8 (31)	<0.001
CV and stroke deaths	0.3 (1)	5.1 (18)	26.3 (93)	<0.001

BP = blood pressure; eGFR = estimated glomerular filtration; HDL: high-density lipoproteins; LDL: low-density lipoproteins; ePWV = estimated pulse wave velocity.

**Table 2:** All-cause and cardiovascular death survival analysis—differences between ePWV terciles according to Kaplan–Meier procedure.

	All-Cause Death Survival Analysis			CV Death Survival Analysis		
Tercile	Average Survival Time (Years)	95% CI	*p* (Log Rank, Mantel–Cox)	Average Survival Time (Years)	95% CI	*P* (Log Rank, Mantel–Cox)
1	18.2 ± 0.1	18.0–18.4	<0.001			0.013
2	17.3 ± 0.2	16.9–17.8		14.0 ± 0.6	12.8–15.2	
3	14.1 ± 0.3	13.5–14.7		11.9 ± 0.4	11.2–12.6	

**Table 3:** Hazard ratios and 95% confidence intervals for cardiovascular mortality in the general population and in subgroups with high cardiovascular risk (Cox regression models).

	Whole Group—General Population					Subgroup with High CV Risk			
		b	*p*	HR	95% CI	b	*p*	HR	95% CI
Model 1	ePWV	0.135	0.038 *	1.145	1.008–1.301	0.269	<0.001 ***	1.309	1.147–1.494
	Ct	−6.181	<0.001 ***	0.000052	0.000007–0.000409	−7.116	0.108	0.000812	1.394–4.7290
	HR	0.012	0.366	1.012	0.988–1.041	0.	0.358	1.013	0.998–1.041
Model 2	ePWV	0.209	0.725	1.232	0.385–3.938	1.108	0.029 *	3.030	1.118–8.211
	Ct	−8.750	0.048	0.000158	0.000158–0.22666	−9.235	<0.001 ***	0.000089	0.000011–0.000754
	Age	0.004	0.972	1.004	0.821–1.226	−0.134	0.129	0.875	0.736–1.040
	Sex	0.057	0.814	1.059	0.659–1.701	0.057	0.803	1.059	0.677–1.657
	MBP	−0.030	0.469	0.970	0.894–1.053	−0.075	0.041	0.928	0.864–0.997
	BMI	−0.019	0.520	0.981	0.925–1.041	−0.054	0.038	0.948	0.901–0.997
	HR	0.020	0.152	1.020	0.993–1.048		0.570	1.008	0.980–1.036
Model 3	ePWV	0.135	0.038 *	1.145	1.007–1.301				
	Ct	0.135	<0.001 ***	0.000044	0.000005–0.000400				
	Diabetes	0.080	0.742	1.083	0.673–1.742				
	HR	0.008	0.591	1.008	0.980–1.036				

* *p* < 0.05; *** *p* < 0.001. Model 3 has not been analyzed for the subgroup with high CV risk, since the patients with diabetes are in a category of high CV risk.

**Table 4:** Hazard ratios and 95% confidence intervals for all-cause mortality in general population (Cox regression model).

Model 2		b	*p*	HR	95% CI
	ePWV	0.643	0.001 ***	1.902	1.282–2.823
	Ct	0.135	0.031 *	0.000007–0.000409	1.349–4.7283
	Age	−0.079	0.011 **	0.924	0.869–0.982
	Sex	−0.110	0.443	0.896	0.676–1.187
	MBP	−0.050	0.001 ***	0.951	0.924–0.979
	BMI	−0.049	0.003 *	0.953	0.923–0.983
	HR	0.015	0.080	1.016	0.998–1.033

* *p* < 0.05; ** *p* < 0.01; *** *p* <0.001.

## Data Availability

All data generated or analyzed during this study are included in this article. Future enquiries can be directed to the corresponding author.

## Supplementary Materials

The following supporting information can be downloaded at https://www.mdpi.com/article/10.3390/jcm13123377/s1, Table S1. Demographic and clinical characteristics of the whole group. Table S2. Laboratory data of the whole group. Table S3. Causes of death in the whole population and in groups of subjects classified in terciles of ePWV. Table S4. Univariate logistic regression analysis—predictors of all-cause mortality. Table S5. Univariate logistic regression analysis—predictors of cardiovascular mortality. Table S6. Causes of death according to diagnoses in the entire population and in groups of subjects classified in terciles according to the ePWV values. Table S7. Characteristics of arterial compliance according the ePWV terciles. Table S8. Results obtained from survival receiver operating characteristic curve identifying the best threshold of ePWV for all-cause, cardiovascular and non-cardiovascular mortality.
