# Supplementary material for: Estimated Pulse Wave Velocity and All-Cause and Cardiovascular Mortality in the General Population
Source: J Clin Med. 2024 Jun 7;13(12):3377. doi: 10.3390/jcm13123377 (PMC11204842; doi:10.3390/jcm13123377)
Supplement: Supplementary file 1 [file jcm-13-03377-s001.zip › jcm-2987442-supplementary.pdf]

SUPPLEMENTARY MATERIALS:

Table S1. Demographic and clinical characteristics of the whole group

	Whole group	Men	Women	P
%	100	42.4	57.6	-
Age (years)	53 ± 16	52 ± 16	54 ± 16	0.03
Systolic BP home visit (mmHg)	137 ± 22	138 ± 20	136 ± 23	0.1
Diastolic BP home visit (mmHg)	84 ± 10	85 ± 10	83 ± 10	0.04
Mean BP home visit (mmHg)	105 ± 14	106 ± 13	104 ± 15	0.074
Heart rate home visit (bpm)	73 ± 9	72 ± 9	74 ± 9	0.01
Systolic BP outpatient visit (mmHg)	135 ± 21	136 ± 19	134 ± 22	0.09
Diastolic BP outpatient visit (mmHg)	83 ± 10	84 ± 10	83 ± 10	0.02
Mean BP outpatient visit (mmHg)	105 ± 13	106 ± 12	104 ± 14	0.04
Heart rate outpatient visit (bpm)	73 ± 9	72 ± 9	74 ± 9	0.05
ePWV outpatient visit (m/s)	9.59 ± 2.52	9.51 ± 2.36	9.65 ± 2.64	0.3
Weight (kg)	77.5 ± 14.8	84 ± 13.5	72.8 ± 13.8	<0.001
Height (cm)	168.8 ± 9.6	176.2 ± 7.5	163.3 ± 6.8	<0.001
Body mass index (BMI) (kg/m^2)	27.2 ± 4.8	27.0 ± 4.0	27.4 ± 5.4	0.2
BMI <25 (%)<br>BMI 25-30 (%)<br>BMI ≥30 (%)	34.4<br>40.8<br>24.8	32.3<br>46.4<br>21.3	35.9<br>36.7<br>27.3	0.005
Hypertension (%)	46.9	47.2	46.6	0.9
Diabetes (%)	9.4	10.4	9.7	0.3
Dyslipidemia (%)	20.2	19.3	20.9	0.7
Chronic kidney disease (%)	20	14.4	23.9	0.016
Previos stroke(%)	1.4	1.7	2.1	0.36
Previous myocardial infarction (%)	2.5	3.9	1.3	0.005
Smokers<br>Current, (%)<br>Ex, (%)<br>Never, (%)	 29.0<br>12.3<br>58.6	 40.5<br>19.7<br>39.7	 21.0<br>7.2<br>71.8	<0.001
Alchohol consumption - never %	55.3	31.4	72.0	<0.001
Physical activity<br>Never/none (%)<br>Sometimes (%)<br>1x per week (%)<br>2x per week v<br>>2x per week (%)<br>Actively (%)	 33.6<br>36.6<br>4.0<br>5.5<br>18.7<br>1.7	 21.1<br>40.0<br>6.2<br>5.7<br>24.1<br>3.0	 42.3<br>34.2<br>2.5<br>5.3<br>14.9<br>0.8	<0.001
Education (years)<br>8 (%)<br>8-12 (%)<br>>12 (%)	 36.0<br>48.3<br>15.7	 24.7<br>54.2<br>21.1	 44.3<br>44.0<br>11.8	<0.001
Monthly income (Eu)<br><200 (%)<br>200-400 (%)<br>400-700 (%)<br>>7000 (%)	 39.4<br>37.7<br>18.4<br>4.5	 27.9<br>41.8<br>22.9<br>7.5	 48.0<br>34.6<br>15.1<br>2.3	<0.001

Values are expressed as mean ± SD, or as percentage (%) ; ; BP = blood pressure

Table S2. Laboratory data of the whole group

	Whole group	Men	Women	p
Serum creatinine (µmol/L)	87 ± 29	95 ± 26	82 ± 30	<0.001
eGFR (ml/min/1,73 m^2)	75.9 ± 18.5	80 ± 18.2	73.0 ± 18.1	<0.001

Fasting blood glucose, (mmol/L)	5.9 ± 1.8	5.9 ± 1.7	5.8 ± 1.8	0.4
Total cholesterol, (mmol/L)	6.04 ± 1.28	6.00 ± 1.27	6.07 ± 1.29	0.6
HDL cholesterol, (mmol/L)	1.36 ± 0.46	1.23 ± 0.29	1.47 ± 0.54	<0.001
LDL cholesterol, (mmol/L)	3.925 ± 1.178	3.914 ± 1.229	3.934 ± 1.114	0.93
Triyglicerids, (mmol/L)	1.82 ± 1.15	1.95 ± 1.21	1.72 ± 1.11	0.04
Uric acid, (mmol/L)	289 ± 93.1	322.2 ± 75.8	262.8 ± 97.3	<0.001

Values are expressed as mean ± SD, or as percentage (%); eGFR= estimated glomerular filtration rate (CKD Epi)

Table S3. Causes of death in the whole population and in groups of subjects classified in terciles of ePWV

Cause of death	1st tertile	2nd tertile	3rd tertile	The whole group
Carcinomas	6 (54,5)	23 (46)	29 (20,5)	58 (28,7)
CV diseases	0	15 (30)	72 (51,0)	87 (43,06)
Stroke	1 (9,0)	3 (6)	18 (12,7)	22 (10,9)
Dementia *	0	3 (6)	8 (5,6)	11 (5,4)
Trauma	0	1 (2)	3 (2,1)	4 (1,9)
COPD	0	0	5 (3,5)	5 (2,5)
Infections	2 (18,1)	1 (2)	4 (2,8)	7 (3,5)
Suicide	1 (9,0)	0	1 (0,7)	2 (0,99)
Other	1 (9,0)	4 (8)	1 (0,7)	6 (2,9)
In total	11 (5,4)	50 (24,7)	141 (69,8)	202

Values are shown as n (%); CV = CV; COPD = chronic obstructive pulmonary disease; * in this group of causes of death, in addition to dementia, senility, Mb Parkinson, Mb Alzheimer's are included.

Table S4. Univariate logistic regression – predictors of all-caused mortality

Variable	HR (95% CI)	P
ePWV (m/s)	1,643 (1,526-1,769)	**<0,001**
age (years)	1,103 (1,087-1,119)	**<0,001**
Height (cm)	1,008 (1,998-1,018)	0,102
Weight (kg)	0,153(0,032-0,731)	0,019
Body mass index (kg/m^2)	1,047 (1,017-1,078)	**0,002**
Systolic blood pressure (mmHg)	1,031 (1,024-1,039)	**<0,001**
Diastolic blood pressure (mmHg)	1,025 (1,009-1,041)	**0,001**
Mean blood pressure (mmHg)	1,039 (1,027-1,051)	**<0,001**
Heart rate (bpm)	1,021 (1,002-1,039)	0,029
Fasting blood gluose (mmol/l)	1,119 (1,009-1,240)	0,033
eGFR (ml/min/1,73 m^2)	0,967 (0,955-0,988)	**<0,001**
Total serum cholesterol (mmol/l)	1,201(1,025-1,408)	0,023
Uric acid (mmol/l)	1,005 (1,002-1,008)	**0,001**
Ct (ml/mmHg)	0.002 (0.001-005)	**<0.001**

eGFR= estimated glomerular filtration rate (CKD Epi); ePWV = estimated pulse wave velocity
*p<0,005

Table S5. Univariate logistic regression – predictors of cardiovascular mortality

Variable	HR (95% CI)	P
ePWV (m/s)	1,949 (1,717-2,214)	**<0,001**
age (years)	1,148 (1,116-1,180)	**<0,001**
Height (cm)	0,008 (0,001-0,098)	**<0,001**
Weight (kg)	1,001 (0,986-1,016)	0,904
Body mass index (kg/m^2)	1,054 (1,011-1,098)	0,013
Systolic blood pressure (mmHg)	1,041 (1,030-1052)	**<0.001**
Diastolic blood pressure (mmHg)	1,036 (1,014-1,060)	**0,002**
Mean blood pressure (mmHg)	1,053 (1,036-1,071)	**<0,001**
Heart rate (bpm)	1,018 (0,990-1,046)	0,203
Fasting blood gluose (mmol/l)	1,213 (1,072-1,373)	**0,002**
eGFR (ml/min/1,73 m^2)	0,955 (0,936-1,231)	**<0,001**
Total serum cholesterol (mmol/l)	1,185 (0,937-1,498)	0,156
Uric acid (mmol/l)	1,005 (1,001-1,010)	0,010
Ct (ml/mmHg)	0,000044 (0,000005-0,00400)	**<0,001**

eGFR= estimated glomerular filtration rate (CKD Epi); ePWV = estimated pulse wave velocity

*p<0,005

Table S6. Causes of death according to diagnoses in the entire population and in groups of subjects classified in terciles according to the ePWV values

Cause of death	1st tertile	2nd tertile	3rd tertile	The whole group
Stroke	1	3	18	22
Myocardial infarction		3	18	21
Ischemic cardiomyopathy		3	18	21
Heart failure #		4	11	15
Hypertensive or atherosclerotic heart disease without congestion		1	13	14
Dementia *		3	8	11
Sudden death		3	1	4
Lung or bronchial cancer	2	4	3	9
Cancer of the colon, rectum or anus		3	4	7
Kidney or bladder cancer		3	4	7
Infections	2	1	4	7
Generalized atherosclerosis			6	6
Gastric cancer	2	1	2	5
Cancer of the liver, gallbladder or pancreas		2	3	5
COPD			5	5
Lymphomas or leukemias			4	4
A malignant neoplasm without a specified site		2	2	4
Trauma		1	3	4
Ovarian or uterine cancer	1	1	1	3
Breast cancer		3		3
Prostate cancer			3	3
Brain tumor		2	1	3
Valve disease		1	2	3
Aortic dissection/rupture			2	2
Carcinoma of the larynx or maxillary sinus		1	1	2
Skin cancer or melanoma		2		2
Suicide	1		1	2
Bone malignancy	1			1
Atrial fibrillation			1	1
Other	1	4	1	6

Table S7: Characteristics of arterial compliance according the ePWV tertiles

	1st Tertile					2nd Tertile					3rd Tertile				
	Mean	Standard Deviation	Median	Percentile 25	Percentile 75	Mean	Standard Deviation	Median	Percentile 25	Percentile 75	Mean	Standard Deviation	Median	Percentile 25	Percentile 75
Ct (mL/mmHg)	,73	,13	,72	,64	,80	,48	,11	,46	,40	,55	,27	,07	,26	,21	,31

Table S8. Results obtained from survival Receiver Operating Characteristic Curve identifying the best threshold of ePWV for all-cause, cardiovascular and non-cardiovascular mortality

Outcome	Cut off Points m/s	95% CI	AUC	Sensitivity	Specificity
CV mortality	10.38	9.98-10.76	0.86	0.85	0.73
All-cause mortality	10.65	10.27-11.01	0.81	0.74	0.75
Non-CV mortality	10.96	10.58-11.38	0.75	0.56	0.78

AUC indicates area under the curve
